# Professional Acquisition of *M. bovis* in Calabria Region (Southern Italy): A Challenging Case of Osteomyelitis in a Migrant Patient from Bulgaria

**DOI:** 10.1155/2015/794715

**Published:** 2015-07-14

**Authors:** Angela Quirino, Carlo Torti, Alessio Strazzulla, Salvatore Nisticò, Luisa Galati, Giorgio Settimo Barreca, Angelo Giuseppe Lamberti, Giuseppina Berardelli, Maria Pacciarini, Giorgio Gasparini, Vincenzo Pisani, Antonio Gambardella, Maria Carla Liberto, Alfredo Focà

**Affiliations:** ^1^Magna Græcia University, 88100 Catanzaro, Italy; ^2^Giovanni Paolo II Hospital, 88046 Lamezia Terme, Italy; ^3^Istituto Zooprofilattico Sperimentale della Lombardia e dell'Emilia Romagna (IZSLER), 25123 Brescia, Italy

## Abstract

We report herein the first case of a coinfection with *Brucella* spp., *M. bovis*, and *Enterobacter cloacae* in a butcher who moved from Bulgaria to Italy. Molecular typing suggested professional acquisition of *M. bovis* in Italy. So, surveillance and preventive measures need to be implemented.

## 1. Introduction

Both tuberculosis due to* Mycobacterium bovis* (*M. bovis*) and brucellosis are zoonotic infections in humans.* M. bovis*, an acid-fast microorganism belonging to the* Mycobacterium tuberculosis complex*, may infect humans through inhalation, ingestion, contact with mucous membranes, and broken skin. Butchers are at risk of pulmonary infections due to inhalation of aerosol particles during the handling of infected meat and carcasses. Nonpulmonary diseases can also be acquired by drinking unpasteurized milk from infected cattle [1]. A survey carried out in the Calabria region during the period 2011-2012 on 98.8% of cattle herds revealed that only <1% of animals were infected by* M. bovis* [2]. Routes of infections can be heterogeneous and unexpected, making it important to characterize the infecting strains and possible risk factors to implement control strategies. Indeed, in restricted epidemics, strains of* M. bovis* were found to be genotypically identical or different only for a single locus [3–5].

*Brucella melitensis* (*B. melitensis*),* B. abortus*,* B. suis*, and* B. canis* are Gram-negative coccobacilli that infect humans. Occupational brucellosis may be acquired in slaughterhouses and butcher shops, during the handling of meat products, and in milk and dairy product industries [6]. In the Calabria region, 82 cases of human brucellosis have been reported between 2007 and 2009, at an average of 27.3 cases per year, accounting for 15.8% of cases reported in Italy [7].

Herein we report the first case of a triple coinfection with* Brucella* spp. and* M. bovis* together with* E. cloacae* in a 45-year-old Bulgarian migrant.* M. bovis* was acquired in Italy during his work as a butcher as suggested by spoligotyping. Therefore, this case underlines the importance of using molecular methods to track the infection of* M. bovis*, should the suspicion of an epidemiological chain arise to guide preventative strategies. Moreover, the present case strengthens the importance of continuing surveillance and control measures.

## 2. Case Report

A 45-year-old Bulgarian patient came to Italy in the year 2000. In his hometown, he worked as a mechanic but, once in Italy, he had been working as a butcher for 10 years. In June 2011, after an accidental trauma with an open wound during work, swelling appeared in his second finger of the left hand, progressively extending to the wrist. So, he had been treated with several antibiotics not recalled by the patient (a part from levofloxacin at unspecified dosage for ten days) without significant response. Then, a surgical treatment for carpal tunnel syndrome was performed in November 2011. In August 2012, a subcutaneous abscess was drained while the patient was in Bulgaria.

In February 2013, fever started and an appropriate work-up at the Infectious Diseases Unit of “John Paul II” Hospital in Lamezia Terme led to a diagnosis of brucellosis, based on clinical manifestations and a positive direct agglutination reaction (Wright test) for* Brucella* spp. (either* B. abortus* or* B. melitensis*). So, he was treated with doxycycline (100 mg twice daily) plus rifampicin (600 mg once daily) for 2 months. At the end of treatment, a significant improvement was recorded with absence of fever and meningeal signs but Wright test for* Brucella* spp. remained positive (1 : 160 titration). After a few days, the patient was admitted for meningitis with headache, vomiting, and neck stiffness. Magnetic resonance imaging (MRI) of the brain revealed “diffuse hyperintensities in T2 sequences at white matter, with small enhanced nodular areas.” Also, clear cerebrospinal fluid with increased protein and reduced glucose concentrations was demonstrated. So, a diagnosis of neurobrucellosis was made. For this reason, he received ceftriaxone (2 g twice daily) for one month, with voluntary discharge after improvement (May 2013). During hospitalization, Wright test became negative (April 16, 2013) but left wrist and hand still remained swollen.

In August and September 2013, two skin swabs were positive for* Staphylococcus aureus* and tobramycin, linezolid, and levofloxacin at unknown dosages were prescribed for about 30 days, without any improvements.

**Figure 1:**
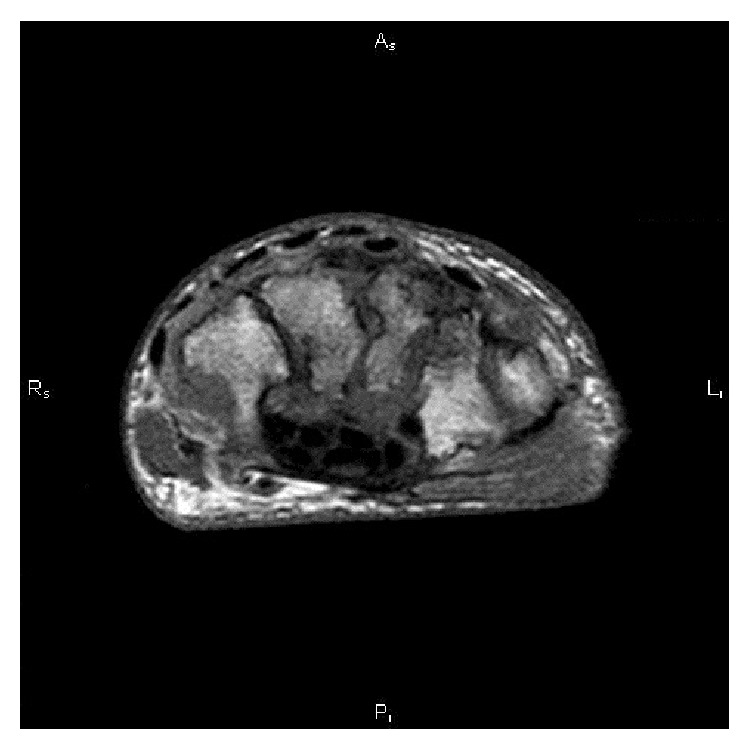
Magnetic resonance imaging of the left wrist before starting antibiotic therapy for* Brucella *spp. and* Mycobacterium bovis*. The picture shows increased signal intensity of trabecular bone of all carpal bones, due to marked medullar edema with many erosive areas, most evident in scaphoid and capitate bones.

The patient presented for consultation at the Infectious Diseases Unit of the University Hospital of Catanzaro in November 2013. On examination, he had a swollen lesion in his left wrist, with a spontaneous drainage from a cutaneous fistula. Also, left knee and the second finger of the right hand appeared swollen. A MRI of the left hand was prescribed, showing “bone lesions attributable to arthritic/septic lesions” (Figure 1).

The patient was then admitted to our day-hospital where further exams were performed: complete blood count (4,900,000 red blood cells/*μ*L, 11,700 white cells/*μ*L with 68.9% neutrophils, and 295,000 platelets/*μ*L) was normal, and also renal and liver function tests appeared to be within the range of normality. Tuberculin skin test (Mantoux test) was positive with an infiltrate of a diameter of 5 cm. Moreover, Quantiferon-TB Gold in-tube (QFT-IT) test was positive. Both* Brucella* spp. DNA from blood (homemade real-time PCR assay as described below) and* Brucella* spp. antibodies (1 : 160 titration) were positive. However,* Brucella* specific treatment was postponed, waiting for confirmation and results of bone biopsy that was planned.

In January 2014, the patient underwent the bone biopsy of the left wrist at the Orthopedics Unit of the University Hospital in Catanzaro, leading to isolation of* Enterobacter cloacae* (*E. cloacae*) spp.* cloacae.* Also, PCR for DNA of* M. tuberculosis complex* (MTBC) and* Brucella* spp. DNA were positive. Afterwards, cultures resulted positive for alcohol-acid resistant bacilli, subsequently characterized as* M. bovis* ssp.* bovis*. Drug susceptibility tests (both genotypic and phenotypic) were performed for rifampicin, isoniazid, ethambutol, streptomycin, pyrazinamide, amikacin, ofloxacin, and linezolid. A molecular line specific probe assay (Genotype MTBRD plus, Hain Lifesciences, GmbH, Germany) was used following manufacturer's instructions. Only resistance to pyrazinamide was detected.

Regarding methods, the samples were processed according to national and international guidelines using an N-acetyl-l-cysteine-NaOH decontamination procedure, inoculated into BACTEC MGIT 960 tubes (Becton Dickinson and Co., Cockeysville, MD, USA) and onto solid slant medium (Löwenstein-Jensen), and incubated at 37°C for up to 4 and 6 weeks, respectively.

MTBC-DNA was amplified with Strand Displacement Amplification (SDA) technology (ProbeTec ET System, Becton-Dickinson and Co., Cockeysville, MD, USA). Amplified MTBC-DNA was revealed only in the soft tissue of the left wrist biopsy. Identification of species was performed using GenoType MTBC assay (Hain Lifesciences, Nehren, Germany). Genotype MTBC assay allowed us to identify the isolate as* M. bovis* ssp.* bovis*. For* Brucella* DNA extraction, either in blood or in bone samples, a Qiagen kit was used following a modified procedure suggested by the manufacturer (Qiagen, Hilden, Germany). For genus-specific real-time PCR assay, the forward primer B4 5′-TGGCTCGGTTGCCAATATCAA-3′ and reverse primer B5 5′-CGCGCTTGCCTTTCAGGTCTG-3′ were used to amplify a 223 bp portion of the* BCSP31* gene [8]. The PCR conditions were denaturation at 95°C for 10 min, followed by 45 cycles (95°C for 10 s, 60°C for 10 s, and 72°C for 9 s). After amplification, melting curve analysis was carried out by evaluating a melting temperature of 88.16 ± 0.05°C.

In February 2014, the patient was admitted to the Neurology Unit of University Hospital of Catanzaro where he underwent brain MRI showing “disseminated foci with signal alteration, most evident in supratentorial areas and medulla oblongata (bulbus).” However, the foci appeared to be reduced in number and size with respect to evaluation performed in April 2013. A lumbar puncture was performed and subsequent cerebrospinal fluid examination showed clear aspect, pH = 8, glucose concentration, erythrocytes, leukocytes, and proteins within the ranges of normality, while cultures and DNA detection for MTBC and* Brucella* spp. were negative.

**Figure 2:**
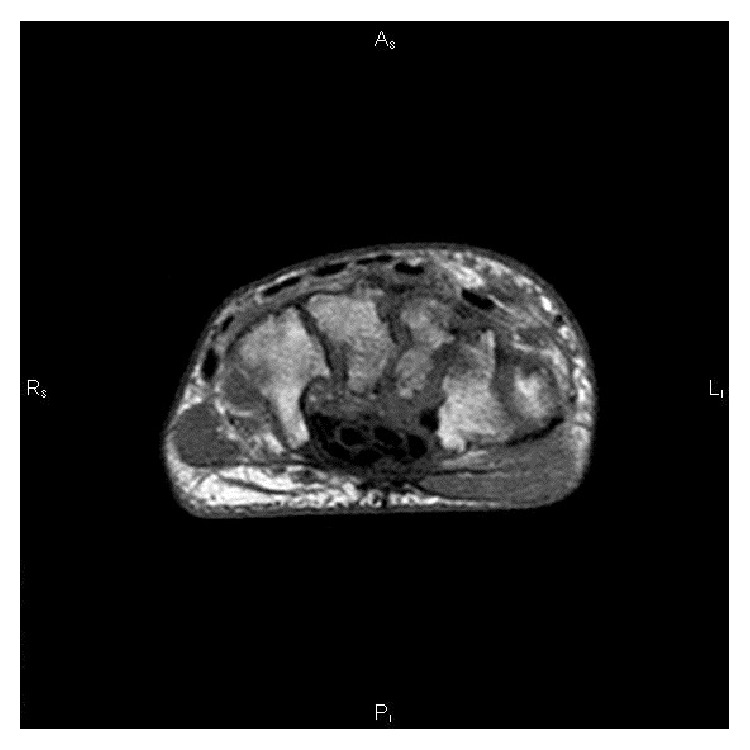
Magnetic resonance imaging of the left wrist after three months of specific antibiotic therapy versus* Brucella *spp. and* Mycobacterium bovis*. The picture shows slight decrease of signal intensity of trabecular bone of radium and carpal bone and moderate reduction of edema extension in scaphoid and capitate bones.

After considering the above findings, from January 30 to February 7, 2014, the patient was treated for* E. cloacae* ssp.* cloacae* infection with ertapenem, 1 g/day intravenously, with a partial recovery of pain and swelling. Then, oral anti-TB therapy was started on February 10, 2014, with isoniazid 300 mg/day plus rifampicin 600 mg/day (with the objective of treating also brucellosis) plus pyrazinamide 2 g/day (interrupted once molecular identification was available showing* M. bovis* naturally resistant to this drug) plus ethambutol 1.6 g/day (interrupted after two months for therapy simplification) plus moxifloxacin 400 mg/day (with the objective of treating also brucellosis). Lastly, on February 24, 2014, intravenous amikacin 1 gr/day plus oral doxycycline 100 mg twice daily was prescribed for treating brucellosis. Amikacin was continued intravenously for 20 days. After 3 months of antibiotic therapy, conditions improved with significant reduction of edema and resolution of functional impairment both in left wrist and in knee. Then, a MRI of left wrist confirmed reduction of the bone lesions (Figure 2).

Interestingly, genomic diversity of* M. bovis* isolated from our patient was assessed and compared to* M. bovis* bovine strains isolated from a cattle herd in the same area around Lamezia Terme. It has to be noticed that this cattle herd provided the animals to the slaughterhouse and butcher's shop where the patient worked, so a transmission chain is further supported. Indeed, by typing of a 24-locus-based mycobacterial interspersed repetitive unit-variable number tandem repeat (MIRU-VNTR) [9], we revealed that just a locus (locus 577) was deleted in the human strain, suggesting epidemiological relatedness. Furthermore, those strains were analyzed by spoligotyping [10] and multilocus variable-number tandem-repeat analysis (MLVA) using 12 markers of VNTR/MIRU: ETRA, ETRB, ETRC, ETRD, and ETRE [11] and VNTR2163a, VNTR2163b, VNTR3155, VNTR4052, VNTR1895, VNTR3232, and MIRU26 [12]. The loci analyzed comprised the 6 loci recommended by the European Network VENoMYC [13]. These molecular typing methods showed that the strains studied were correlated.

## 3. Discussion

We investigated a unique coinfection with* M. bovis*,* Brucella* spp., and* E. cloacae*.* Brucella* spp. infection involved three different sites (blood, brain, and bone) as demonstrated by molecular methods. So, these methods were important for an etiological diagnosis in different body compartments.

For* M. bovis,* molecular analysis strongly suggested that the isolate from our patient belonged to the same chain of transmission of animal strains in the same area and the same period of time. In fact, cattle breeding, slaughterhouse, and butcher's shop were part of a unique facility. In previous studies, mutations frequency was demonstrated to be very low, so genotypes could be strictly correlated with the same transmission chain in animals even after 4-5 years, especially in a geographic restricted area [3–5].

Since no* M. bovis* infections were notified in the slaughterhouse where the patient was working but the isolated strain was related to strains circulating in animals in the same area, the present case indicates that surveillance systems should be implemented, especially in regions where these infections are still endemic.
